# Public Health and Preventive Medicine – “The RED BOOK”

**Published:** 2009-04

**Authors:** Amarjeet Singh

**Affiliations:** Professor of Community Medicine, PGIMER School of Public Health, Chandigarh, India. E-mail- amarminhas56@rediffmail.com

This state-of-the-art book is a rich collection of public health text. It should be available to all and not just restricted to the armed forces. After going through the book I got a feeling that so far I had been seriously missing such a text. At last, my soul can rest in peace that we can also now boast of having a Preventive and Social Medicine (PSM) book, which, in future, may compete for international attention. An extract from Winslow's text has been well chosen as the beginning of the book. The chapter on Epidemiology demonstrates a new way of looking at things. Quoting good real life examples is a welcome step. Statistics and software chapters are also good. The first “unnamed and unnumbered” chapter is the master stroke and is the best part of the book. Mature, original, and clear writing indeed! Very thought provoking! The seemingly unending list of core principles, 12 core activities, and 12 golden rules look refreshingly original. This writeup reflects a deep understanding of the philosophy of our discipline. The basis for chapter organization has also been well explained. However, I wonder, why it was not given a label and a space in the Contents.

It starts with a provocative subtitle, “Do we need Public Health and Preventive Medicine?” Yet, no answer has been provided. Moreover, the rationale of the title of the book has also not been explained. Incidentally, this book shares its title with the classic textbook of Maxcy. Is preventive medicine different from public health (or is it a part of public health or the other way round as the authors claim)? This issue needs to be settled. Although the foreword explains this a little bit, the transformation of “Field Service Hygiene Notes” to “Manual of Hygiene” and then to “Manual of Health” and now to this book needs more clarification. Names and terminology do matter.[1]

Special health issues for the armed forces and military preventive medicine are quite useful. The chapter on Entomology is also good as is the one on Insect-Borne Diseases. Many practical tips are given. Overall, the text of the book is methodical and systematic. Many new things are present, for example, field kit for testing poison in water, environmental health emergencies, desert medicine, and so on. A balanced distribution of different subject areas is present. The chapter on Environmental Health Sciences is quite good, which also dwells on the clinical management as well as preventive measures for clinical conditions.

Having spent my formative years in an army cantonment I had the privilege to experience its clean and green ambience. The daily one hour PT drill in an army school, the clean army mess, fresh air, vast *lungar* halls for *jawans*, ration stores built on a three-feet high, raised, cemented platform, rat proofing and fly proofing, which we are supposed to teach and demonstrate to our undergraduate and postgraduate students, were actually being practiced in the army. But sadly, all these and many more *health promotion* activities including emphasis on physical fitness and cleanliness for which the army is famous, have not been given due importance in the book. The army's strong point is health promotion and not epidemiology. After all, epidemiology is just a tool. The action is embedded in the concept of health promotion. The army provides the evidence of the benefit we can have if we focus on health promotion. Personally, I often teach students that it is the army where preventive medicine including health promotion is truly practiced. Pre-placement examination, regular medical checkups, food hygiene, insect control, sanitation, and sports – all PSM concepts in the real sense – are implemented in the army. Hence, why ignore the same in the text book?

I also wonder why the writers on pubic health have started ignoring history. Some books on PSM, which have hit the market of late, have done just that. On the one hand, this book quotes from Winslow and on the other hand there is nothing in the text about our origin or roots. Neglect of history reflects a deep malady, a lack of conviction about our discipline's worth. Of all the disciplines, public health is the one that should give more emphasis to the context. History puts things in a proper context. History provides us a depth of understanding. History will tell us how and why different terminologies related to public health have emerged.

The book does not elucidate the concept of levels of prevention. The good old Leavell and Clark's beautiful dealing of public health concepts through the “levels of prevention” approach has been ignored. It was quite surprising and puzzling to note that a book on public health and preventive medicine has “Epidemiology” as the first chapter. Apparently, the authors have been swayed by the market forces! Social sciences have not been adequately explained.

By using the word “lifestyle” in the title of the chapter, the authors seem to have fallen prey to the “victim blaming” paradigm and have practically put the blame of non-communicable diseases (NCDs) on individuals. They seem to have ignored the fact that genetic, structural, and policy level factors are also important in the etiology and management of NCDs.

Titles of some diagrams and tables are missing! Toxic shock syndrome has not been mentioned in the measles vaccine text! Plague: The 2004-2005 outbreak in Uttaranchal has not been covered. The difference in surveillance and monitoring has not been explained properly. In many places, the type of language used by the authors interferes with the comprehension of the text.

The price of the book is not mentioned. The book is certainly bulky with 1100 pages and 141 chapters. All said and done, this is an excellent book on public health. It should serve as a challenge as well as a standard against which future authors in our fraternity should sharpen their writing skills.
